# Depletion of Cytotoxic T-Cells Does Not Protect *NUP98-HOXD13* Mice from Myelodysplastic Syndrome but Reveals a Modest Tumor Immunosurveillance Effect

**DOI:** 10.1371/journal.pone.0036876

**Published:** 2012-05-11

**Authors:** Sheryl M. Gough, Yang Jo Chung, Peter D. Aplan

**Affiliations:** Leukemia Biology Section, Genetics Branch, National Cancer Institute, National Institutes of Health, Bethesda, Maryland, United States of America; Escola Paulista de Medicina - UNIFESP, Brazil

## Abstract

Myelodysplastic syndrome (MDS) and aplastic anemia (AA) patients both present with symptoms of bone marrow failure. In many AA patients, these features are thought to result from an oligoclonal expansion of cytotoxic T-cells that destroy haematopoietic stem or progenitor cells. This notion is supported by the observation that AA patients respond to immunosuppressive therapy. A fraction of MDS patients also respond well to immunosuppressive therapy suggesting a similar role for cytotoxic T-cells in the etiology of MDS, however the role of cytotoxic T-cells in MDS remains unclear. Mice that express a *NUP98-HOXD13* (*NHD13*) transgene develop a MDS that closely mimics the human condition in terms of dysplasia, ineffective hematopoiesis, and transformation to acute myeloid leukemia (AML). We followed a cohort of *NHD13* mice lacking the Rag1 protein (*NHD13*/*Rag1*KO*)* to determine if the absence of lymphocytes might 1) delay the onset and/or diminish the severity of the MDS, or 2) effect malignant transformation and survival of the *NHD13* mice. No difference was seen in the onset or severity of MDS between the *NHD13* and *NHD13*/*Rag1*KO mice. However, *NHD13/Rag1*KO mice had decreased survival and showed a trend toward increased incidence of transformation to AML compared to the *NHD13* mice, suggesting protection from AML transformation by a modest immuno-surveillance effect. In the absence of functional Tcrb signaling in the NHD13/*Rag1*KO T-cell tumors, *Pak7* was identified as a potential Tcrb surrogate survival signal.

## Introduction

Myelodysplastic syndrome (MDS) comprises a heterogeneous group of clonal stem cell disorders characterized by ineffective hematopoiesis, peripheral blood cytopenias despite a hypercellular or normocellular bone marrow, and morphologic evidence of dysplasia. MDS has been associated with a number of genetic abnormalities, most commonly unbalanced chromosomal aberrations such as 5q-, -7/7q-, trisomy 8 and 20q- [1], [2] (reviewed in [3], [4]). Mutations in a large number of genes are also associated with MDS and include *ASXL1, DNMT3A, EZH2, IDH1/IDH2, NRAS/KRAS, RUNX1, TET2* and *TP53* (reviewed in [3], [5]). More recently, multiple mutations have been found in genes integral to the spliceosome complex [6], [7], [8]. Although less common, balanced chromosome translocations are also associated with MDS, eg., t(1;3), t(2;11), t(10;12) and t(11;16) (November 2011[9]).

Similar to MDS, aplastic anemia (AA) is also characterized by peripheral blood cytopenias. However, AA is associated with a hypocellular bone marrow and is thought to be caused in the majority of cases by an oligoclonal expansion of cytotoxic T-cells that target and destroy hematopoietic stem and progenitor cells, resulting in a lack of mature, functional hematopoietic cells [10]. Auto-immune mediated destruction of hematopoietic stem and progenitor cells has also been proposed to play a role in some cases of MDS, (reviewed in [4]), and this assertion is supported by the observation that a subset of MDS patients respond to immunosuppressive therapy (reviewed in [11], [12]). Additional evidence for an autoimmune role in a subset of MDS patients includes the expansion of clonal cytotoxic TCR-V*β* T-cell populations which are depleted after immunosuppressive therapy [13], [14], [15], [16], [17], the preferential response of patients with specific human leukocyte antigen (HLA) subtypes, and increased levels of selected pro-inflammatory cytokines (TNFα, IFNγ) in the bone marrow (reviewed in [11], [12]).

The fusion gene *NUP98-HOXD13* results from the translocation t(2;11)(q31;p15) and has been detected, albeit only rarely, in MDS as well as AML patients [18]. Although this translocation is rare in patients with MDS, it leads to overexpression of *HOXA* cluster genes, especially *HOXA7* and *HOXA9*, which is a common finding in patients with MDS [19], [20]. Transgenic mice that express a *NUP98-HOXD13* (*NHD13*) fusion gene have previously been shown to develop a MDS that closely resembles human MDS, with ineffective hematopoiesis, peripheral blood cytopenia, morphologic dysplasia, increased apoptosis, and transformation to acute leukemia between 8 and 14 months of age [21]. *Rag1* deficient mice lack mature T- and B-lymphocytes, due to their inability to recombine immunoglobulin and T cell receptor genes [22]. Therefore, to test the hypothesis that a lack of cytotoxic T-lymphocytes will provide a protective effect against the onset and/or severity of the MDS that develops in the *NHD13* mice, we crossed the *NHD13* transgene onto a *Rag1* deficient (*Rag1*KO) background.

## Materials and Methods

### Ethics Statement

Animal experiments were approved by the National Cancer Institute Animal Care and Use Committee.

### Transgenic Mice

C57BL/6 *NHD13* [21] mice were bred with B6;129S7-*Rag1* ^tm1Mom^/J (*Rag1* ^−/−^) mice [22] (Jackson Laboratories, Maine, USA). The mating strategy involved crossing the C57BL/6 *NHD13* ^+/−^ to B6;129S7-*Rag1* ^tm1Mom^/J mice to produce *NHD13^+/−^Rag1^+/−^* and *NHD13^−/−^Rag1^+/−^* progeny. *NHD13^+/−^Rag1^+/−^* mice were then backcrossed to the B6;129S7-*Rag1* ^tm1Mom^/J mice to generate the study cohorts *NHD13* ^+/−^ *Rag1* ^+/−^, *NHD13^+/−^Rag1* ^−/−^, *NHD13^−/−^Rag1* ^+/−^, and *NHD13^−/−^Rag1* ^−/−^. Mice were housed in a Specific Pathogen-Free (SPF) environment. Genomic DNA isolated from tail biopsies were used to genotype mice using polymerase chain reaction (PCR) primers as previously described [21] (for *NHD13*) or recommended by Jackson Labs (*Rag1*).

### Evaluation of Mouse Health

Mouse health was monitored by serial complete blood counts (CBCs) and observation. Tail vein peripheral blood was collected in EDTA-tubes and CBCs were determined every two months using a HEMAVET Multispecies Hematology Analyzer (CDC Technologies). Each cohort was followed to determine course of MDS, potential transformation to leukemia and cause of death. Mice were euthanized for necropsy and analysis if CBCs indicated leukemic transformation or severe anemia (Hemoglobin <6 grams/dL). Mice were also euthanized if they displayed non-specific signs of illness such as weight loss, lethargy, kyphosis, dyspnea, or were moribund. Upon necropsy, the presence or absence of splenomegaly, hepatomegaly or enlarged thymus in the leukemic mice was noted. Diagnoses were determined using a combination of CBCs, necropsy findings, morphology, histology, immunohistochemistry, and flow cytometric analysis of bone marrow, spleen, and thymus if appropriate. Mice were classified according to the Bethesda proposals for non-lymphoid and lymphoid neoplasms in mice [23], [24].

### Flow Cytometry

Single cell suspensions in Hank’s balanced salt solution (HBSS, Invitrogen) with 2% fetal bovine serum (HF2) were stained with fluorophore-conjugated antibodies (eBiosciences or BD Pharmingen) and incubated on ice for 45 mins. Following staining, cells were washed with PBS and re-suspended in HF2 containing propidium iodide [1 µg/ml] (Sigma), and analysed with a five laser FACScan (Becton Dickinson).

### Immunohistochemistry (IHC)

Tissue sections and hematoxylin-eosin (H&E), myeloperoxidase (DAKO), CD3 (AbD Serotec) and B220/CD45R (PharMingen) staining, were performed by the Pathology/Histotechnology Laboratory (NCI-Frederick) using conventional staining techniques, on tissue fixed in 10% NBF. Air dried peripheral blood smears and cytospins of single cell suspensions were stained with May-Grünwald-Giemsa for morphological evaluation.

### Southern Blot Analyses

Genomic DNA (10 µg) was digested with both *Hin*dIII and *Sst*I independently, separated on a 0.8% agarose gel and transferred to nylon membranes (Genescreen Plus Hybridisation Transfer Membrane, Perkin Elmer, Boston MA). Membranes were hybridised with a ^32^P labelled *TCRB* probe (Ready-To-Go DNA Labelling Beads (-dCTP), Amersham GE Healthcare, UK) to detect *Tcrb1* and *Tcrb2* constant regions. Results were detected using BioMax XAR film (Kodak).

### RQ-PCR Mouse Apoptosis Gene Expression Arrays

RNA was isolated from wild type control thymus, *NHD13* and *NHD13/Rag1*KO thymic tumours using a standard Trizol/chloroform protocol (Invitrogen). Reverse transcriptase quantitative PCR (RQ-PCR) was used to assay selected genes involved in apoptosis using a kit from SABiosciences. Briefly, cDNA was synthesised using the RT^2^ First Strand Kit (C-03), and RT^2^ Profiler™ PCR Array Mouse Apoptosis plates (PAMM-012) were set up with RT^2^ SYBR Green/ROX qPCR Master Mix (PA-012). Data analysis was performed using SABiosciences RT^2^ Profiler PCR Array Data Analysis software (http://pcrdataanalysis.sabiosciences.com/pcr/arrayanalysis.php).

### Statistical Analyses

Graphed data are represented as the mean ± SEM where applicable. Statistical significance is calculated using students T-Test. Mantel-Cox tests were used to determine statistical significance between survival cohorts, and Two-way Anova for the MDS and leukemia frequencies in *NHD13* vs *NHD13/Rag1*KO cohorts.

## Results

### Lymphocyte Depletion does not Protect *NHD13* Mice from Developing MDS

**Figure 1:**
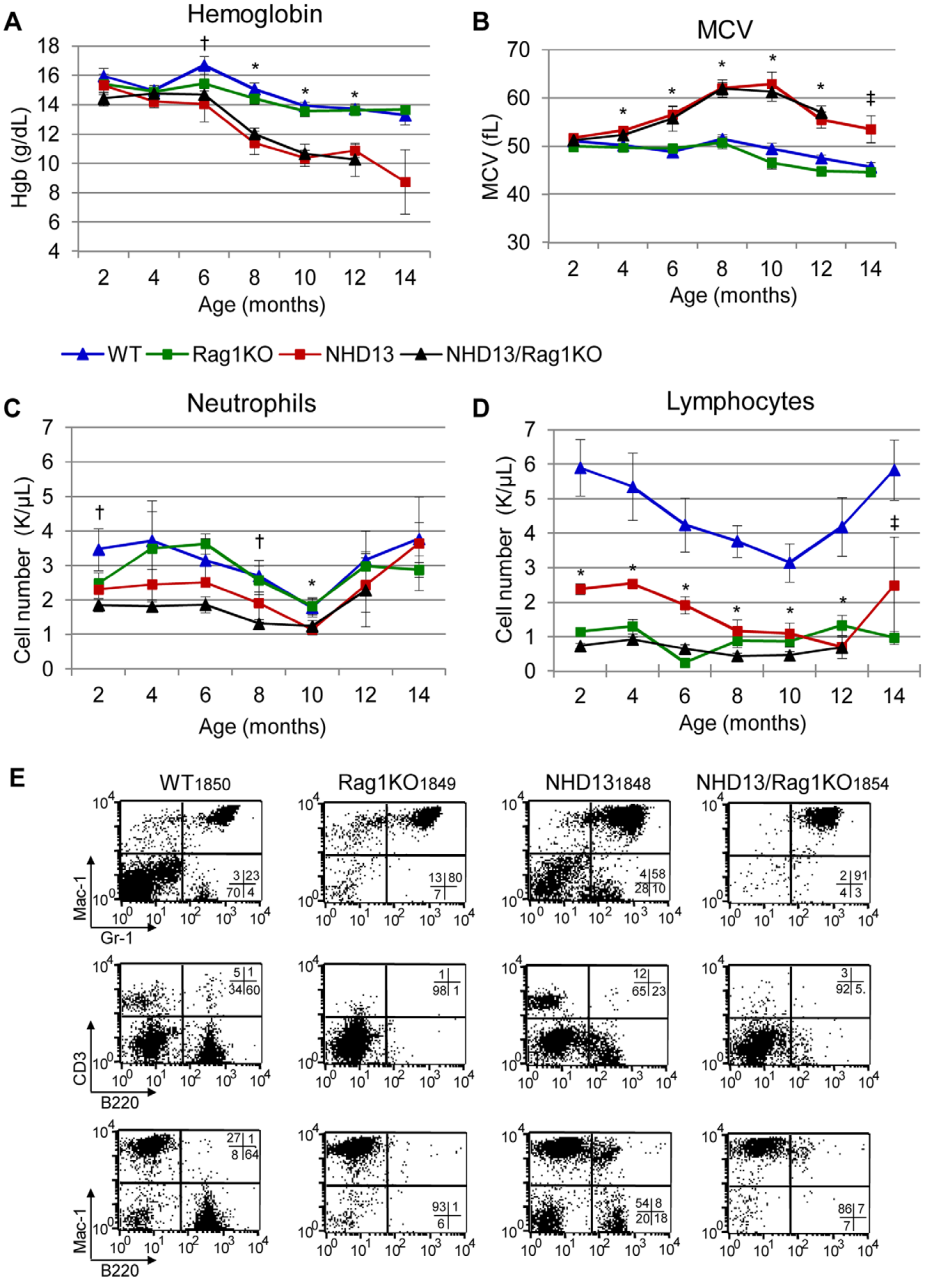
Peripheral blood (PB) indices are similar in *NHD13* and *NHD13/Rag1*KO mice. Hemoglobin levels (A) are decreased and MCV levels (B) are increased in *NHD13* and *NHD13/Rag1*KO mice; the decline in MCV at 12 and 14 months is likely due to death of the more severely affected mice. (C) and (D) Neutrophil and lymphocyte counts; results in C and D exclude mice that have transformed to leukemia (WBC >20 K/µL). Error bars in A-D represent the standard deviation. Timepoints with p values <0.05 are indicated as follows; (*) indicates significance for both *NHD13* vs WT and *NHD13/Rag1*KO vs WT comparisons, (†) *NHD13/Rag1*KO vs WT only, and (‡) *NHD13* vs WT only. The number of mice at each time point varies as mice die over time (Table 1). (E) Representative FACS analysis of PB from mice of each genotype, aged 8 months, stained with the indicated antibodies.

To test the hypothesis that a lack of mature lymphocytes, including cytotoxic T cells, would protect *NHD13* transgenic mice from developing MDS, we crossed the *NHD13* transgene onto a *Rag1* ^−/−^ background. Given that one copy of the *Rag1* gene is sufficient to produce normal levels of lymphocytes [22], *Rag1* ^+/−^ mice were regarded as wild-type (WT) with respect to *Rag1*. Serial CBCs were obtained every other month to determine if the mice had developed evidence of MDS such as pancytopenia or dysplasia. *NHD13* ^−/−^/*Rag1* ^+/−^ (hereafter WT) and *NHD13* ^−/−^/*Rag1* ^−/−^ (hereafter *Rag1*KO) had hemoglobin levels that were indistinguishable from one another, and 3–4 g/dl greater than *NHD13* ^+/−^/*Rag1* ^+/−^ (hereafter *NHD13*) and *NHD13* ^+/−^/*Rag1* ^−/−^ (hereafter *NHD13/Rag1*KO) (Figure 1A, Table 1, *[NHD13 vs WT and NHD13/*Rag1*KO vs WT] †[NDH13/*Rag1*KO only vs WT] indicates timepoints with p = <0.05, t-test). The mean corpuscular volume (MCV) of the *NHD13* and *NHD13/Rag1*KO were consistently elevated compared to WT mice, but not different from one another (Figure 1B). Taken together, these findings indicate that the *Rag1* status does not affect the macrocytic anemia which develops in the *NHD13* mice [21], [25].

**Table 1:** Number of mice evaluated by serial CBC.*

	Age (months)			
Genotype	8	10	12	14
*NHD13*	17	13	9	4
*NHD13/Rag1*KO	24	15	7	0
WT	14	14	11	11
*Rag1*KO	13	13	10	11

* mice transformed to leukemia are not included.

As expected, the absolute neutrophil count (ANC) was normal and the absolute lymphocyte count (ALC) was decreased in the *Rag1*KO compared to WT, with an ALC of ∼1000/uL, compared to WT controls of ∼5000/uL (Figure 1C,D). However, an ALC of even 1000/uL in *Rag1*KO mice is somewhat surprising, since these mice should have no B or T cells. To verify that the *Rag1*KO deficiency was not “leaky”, we assayed peripheral blood mononuclear cells (PBMCs) for the presence of B220 (a marker of mature B cells) and CD3 (a marker of mature T cells) (Figure 1E). Neither of these antigens was expressed in the PBMCs from *Rag1*KO mice or *NHD13/Rag1*KO mice, indicating that the knockout was not “leaky” with respect to T and B lymphocytes, and that the lymphocytes detected by the Coulter counter were likely to be NK cells or small monocytes. As previously described [26], [27], the *NHD13* mice had decreased ANCs and ALCs (Figure 1C,D, ‡p = <0.05, t-test), compared to WT. The *NHD13/Rag1*KO showed similar differences compared to WT (Figure 1C,D, †p = <0.05, t-test), however there was no improvement in ANC levels and further reduced ALCs compared to the *NHD13* mice. Both the *NHD13* and the *NHD13/Rag1*KO mice had an unusual Mac-1^+^/B220^+^ population (Figure 1E, bottom row). This Mac-1^+^/B220^+^ population has been previously identified in mice expressing the *NUP98-HOXD13* [25], *NUP98-PHF23* (SG and PDA, unpublished) and *CALM-AF10* [28], [29] fusion genes.

In addition to the peripheral blood cell counts, there were no obvious differences in morphologic signs of bone marrow dysplasia, such as hypersegmented neutrophils, nuclear bridging, multinucleate cells and blast counts), between the *NHD13/Rag1*KO and *NHD13* mice (data not shown). Thus, the absence of lymphocytes, including cytotoxic T-cells, had no effect on the onset or severity of MDS in this mouse model, suggesting that cytotoxic T-cells do not play a role in the etiology of MDS in the *NHD13* mouse model.

### *NHD13/Rag1KO* Mice Show a Reduced Survival and Earlier Transformation to Acute Leukemia

**Figure 2:**
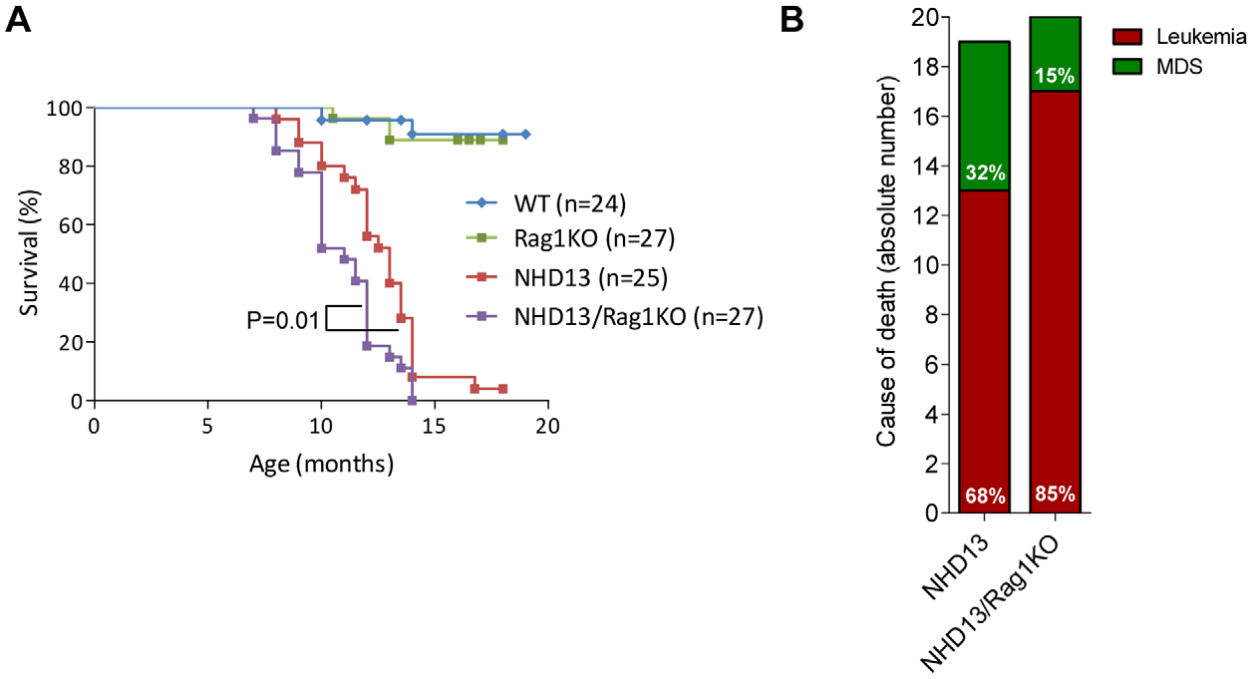
*NHD13/Rag1*KO mice have decreased survival and an increased incidence of leukemia. (A) Survival is dramatically reduced in the *NHD13* mice and further decreased in the *NHD13/Rag1*KO cohort (Mantel Cox test p = 0.01); median lifespan of the *NHD13/Rag1*KO mice is two months less than the NHD13 cohort. (B) Transformation to leukemia was 25% higher in the *NHD13/Rag1*KO mice compared with *NHD13*. Twenty-five percent and 26% of mice were found dead in the *NHD13* and *NHD13/Rag1*KO cohorts respectively, and were unavailable for necropsy, therefore these mice were excluded from the analyses in panel B.

The *NHD13/Rag1*KO mice demonstrated reduced survival compared with the *NHD13* mice, and all the *NHD13/Rag1*KO mice were dead by 14 months of age (Figure 2A, Mantel-Cox, P = 0.01). Median survival of the *NHD13* cohort was 13 months whereas the *NHD13/Rag1*KO cohort had a median survival of 11 months. The *NHD13* mice were less likely to transform to acute leukemia than were the *NHD13/Rag1*KO mice (13/19 vs. 17/20), and more likely to die with progressive MDS (6/19 vs 3/20) (Figure 2B), although these differences did not reach statistical significance (P = 0.20, Two-way ANOVA). Death from severe MDS or leukemia was not associated with any age biases.

**Figure 3:**
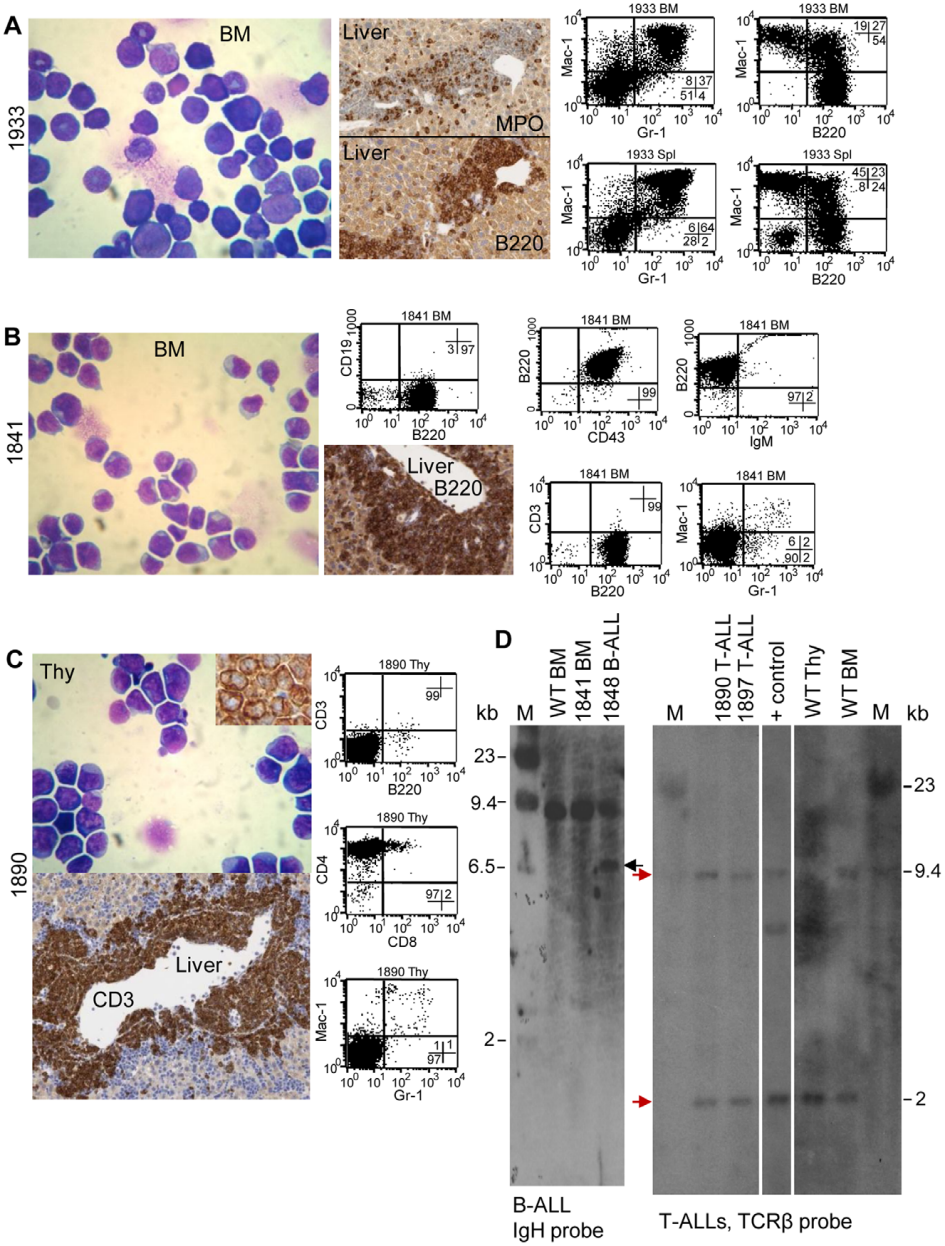
Representative leukemias in *NHD13/Rag1*KO mice. (A) Representative AML (#1933) May-Grunwald/Giemsa stained cytospin shows blasts and immature neutrophils in the bone marrow (left panel). Perivascular infiltration of MPO^+^/B220^+^ cells is shown in the liver (middle panels) and a Mac-1^+^Gr-1^+^B220^+^ population dominates both the bone marrow and spleen tissues (FACS plots, right). (B) Representative B-ALL (# 1841). Bone marrow replaced with lymphoblasts (left panel). Perivascular infiltration of B220^+^ cells in the liver is evident (middle panel), and FACS (right) shows the abnormal population to be B220^+^CD43^+^CD19^-^SIgM^-^CD3^-^Mac-1^-^Gr-1^-^. (C) Representative T-ALL (#1890). Cytospin reveals sheets of lymphoblasts; inset shows cytoplasmic CD3 staining of thymic blasts. Perivascular infiltration of CD3^+^ cells is shown in the liver (lower panel). FACS (right) revealed a CD3^−/^CD4^+^ cell population in the thymus. (D) Southern hybridization with IgH (left panel) and TCRb (right panel) probes. Left panel, WT and 1841 BM shows germline IgH genomic configuration. 1848 B-ALL is a representative *Rag1* competent *NHD13* mouse with B-ALL, showing clonal IgH gene rearrangement (black arrow). Right panels show 1890 and 1897 T-ALL genomic DNA with the TCR locus in germline configuration (red arrows). Wild-type thymus shows expected loss of the CB1 fragment, and wild type bone marrow (WT BM) demonstrates germline TCR configuration. + control, TCR rearranged positive control DNA from the same Southern blot. M, λ *Hin*dIII ladder.

Previous studies [21], [30] have demonstrated that the *NHD13* mice develop a range of leukemias, including erythroleukemia, AML, T-cell ALL, and B-cell ALL. Somewhat surprisingly, given that the *NHD13/Rag1*KO mice cannot generate T or B lymphocytes due to a lack of VDJ recombination, the frequency of lymphoid leukemias were similar in both the *NHD13/Rag1*KO mice (5/20) and the *NHD13* mice (6/19) (Table 2). Three representative *NHD13/Rag1*KO leukemia subtypes are shown in Figure 3; Figure 3A shows an example of AML. The CBC revealed anemia with a hemoglobin of 3.0 g/dL, an elevated MCV of 72 fL, an elevated WBC of 46,000/µL, and a low platelet count of 95,000/µL. The bone marrow was infiltrated with sheets of myeloblasts, and parenchymal organs, including the spleen and liver, were infiltrated with leukemic blasts. The cells that invaded the liver stained for both myeloperoxidase and B220, and FACS analysis demonstrated that the leukemic cells were predominantly Mac-1^+^/Gr-1^+^. Of note, many of these Mac-1^+^/Gr-1^+^ cells were also positive for B220; a Mac-1^+^/Gr-1^+^/B220^+^ population has been observed previously in AML caused by overexpression of the *CALM-AF10* [28], [29]or *NUP98-HOXD13* [25] fusion genes in mice, and a similar lymphoid-primed multipotential progenitor population has recently been reported in a subset of human AML samples [31]. Interestingly, some of the malignant cells seem to have lost Mac-1 and Gr-1 expression, and express only a B220 “dim” population (Figure 3A); this phenomenon has been noted previously with AML caused by a *CALM-AF10* fusion. In that setting, the B220 dim cells were enriched for leukemia stem cells [28].

**Table 2:** Summary of Diagnoses.

No.	*NHD13*	Age/month		Diagnosis		No.	*NHD13/Rag1*KO	Age/month		Diagnosis	
1	A1839	10		MDS		1	A1838	12		MDS	
2	A1855	13.5		MDS		2	A1910	13.5		MDS	
3	A1877	14.5		MDS		3	A1929	12		MDS	
4	A1888	14		MDS		4	A1854	11		AML	
5	A1918	13.5		MDS		5	A1856	12		AML	
6	A1962	17		MDS		6	A1860	10		AML	
7	A1851	10		AML		7	A1896	14		AML	
8	A1895	12		AML		8	A1907	14		AML	
9	A1898	14		AML		9	A1914	13		AML	
10	A1917	13		AML		10	A1916	8		AML	
11	A1921	14		AML		11	A1933	11.5		AML	
12	A1971	8		AML		12	A1943	12		AML	
13	A1967	18		AML*		13	A1950	10		AML	
14	A1848	12		B-ALL		14	A1951	12		AML	
15	A1904	12.5		B-ALL		15	A1841	14		B-ALL	
16	A1927	11.5		B-ALL		16	A1926	12		B-ALL	
17	A1931	12		B-ALL		17	A1966	11.5		B-ALL	
18	A1853	11		T-ALL		18	A1890	10		T-ALL	
19	A1915	13		T-ALL		19	A1897	10		T-ALL	
20	A1837	13.5		FD/unknown		20	A1894	7		Leukemia NOS**	
21	A1862	12		FD/unknown		21	A1842	10		FD/unknown	
22	A1922	14		FD/unknown		22	A1861	8		FD/unknown	
23	A1924	13		FD/unknown		23	A1902	10		FD/unknown	
24	A1938	9.5		FD/unknown		24	A1925	10		FD/unknown	
25	A1953	9		FD/unknown		25	A1940	9		FD/unknown	
						26	A1952	9		FD/unknown	
						27	A1961	8		FD/unknown	

* Euthanised at end of study – mouse asymptomatic although CBC at euthanasia showed elevated WBCs (24.78 K/µL) and neutrophils (19.52 K/µL). Splenomegaly observed upon necropsy. H&E showed perivascular infiltration of the liver with invading cells approximately 50% MPO+ by IHC. FACS showed BM 54% Mac-1^+^/Gr-1^+^, 40% Ter119^+^ and spleen 25% Mac-1^+^/Gr-1^+^, 75% Ter119^+^.

** NOS, not otherwise specified – mouse found dead, with hepatosplenomegaly. H&E showed extensive parenchymal invasion of blasts in the liver, spleen and kidney. IHC shows invasive cells to be B220-, CD3- and MPO-.

An example of B-ALL (mouse 1841) is shown in Figure 3B. The CBC from this mouse revealed a hemoglobin of 9.1 g/dL, an elevated MCV of 60 fL, an elevated WBC of 61.32 K/µL, and a normal platelet count at 998 K/µL. The bone marrow was replaced by sheets of lymphoblasts and examination of the liver showed a perivascular infiltration of B220^+^ cells. In contrast to the AML shown above, these cells were MPO negative. These findings were consistent with the FACS analysis which demonstrated a homogeneous B220^+^/CD43^+^/CD19^−/^IgM^-^ cell population in the bone marrow (shown), indicative of an immature B-cell phenotype, and negative staining for the myeloid antigens Mac-1 and Gr-1. FACS analysis and IHC of the spleen and cells that had infiltrated the thymus showed a similar pattern (not shown). Figure 3C shows findings from a mouse with T-ALL (mouse 1890). The CBC from this mouse revealed a hemoglobin of 10.6 g/dL, an elevated MCV of 65 fL, an elevated WBC of 36,500/µL, and a normal platelet count at 857,000/µL. The normal thymic architecture was effaced and replaced by sheets of lymphoblasts. IHC demonstrated cytoplasmic CD3 staining in the thymus, and the liver showed perivascular invasion of CD3^+^ cells. Of note, despite the fact that IHC was strongly positive for CD3, FACS analysis demonstrated that the cells were CD3^-^CD4^+^, and negative for Mac-1 and Gr-1.

To determine whether the lymphoid cell that is a target for leukemic transformation is a rare cell that has undergone a functional *Igh* or *Tcrb* gene rearrangement despite the lack of Rag1 protein, as is the case with *scid* mice that develop pre-T-ALL[32], we searched for evidence of clonal *Igh* or *Tcrb* gene rearrangements in the B and T cell leukemias, respectively. As shown in figure 3D, the B- and T-cell malignancies retained the germline configuration of *Igh* and *Tcrb*, respectively. The absence of a clonal *Tcrb* gene rearrangement now helps explain why the leukemic cells from mouse #1890 were positive for cytoplasmic CD3, but negative for surface CD3. Tcrb is required for the CD3/TCR complex to form in the ER and delivery of the CD3/TCR complex to the plasma membrane [33], [34]. Since *NHD13/Rag1*KO mice do not produce Tcrb, but can produce CD3, the CD3 protein therefore remains in the ER.

### Pak7 May Replace Tcrb as a T-cell Survival Signal in the *NHD13/Rag1KO* Mice

The development of lymphoid leukemias was an unanticipated finding in the *NHD13/Rag1*KO mice. During normal thymocyte differentiation, positive selection via the TCR ensures T-cell rescue from apoptosis and directs further differentiation to the CD4/CD8 double positive stage (reviewed in [35], [36]). We have previously demonstrated that *scid* mice, which are also defective in VDJ joining, are relatively protected against the oncogenic effects of *SCL* and *LMO1* transgenes, and that the T-ALLs that do develop in these mice universally have an in-frame *Tcrb* gene rearrangement, suggesting that the rare “leaky” *scid* thymocyte with a functional *Tcrb* is the target for malignant transformation. However, the *Rag1*KO is not leaky, and we saw no evidence of *Tcrb* gene rearrangements in the T-ALLs that arose in the *NHD13/Rag1*KO mice. Therefore, in the absence of T-cell receptor signaling, it is reasonable to predict that an alternative survival signal may be functional in the *NHD13/Rag1*KO T-cell ALLs.

**Figure 4:**
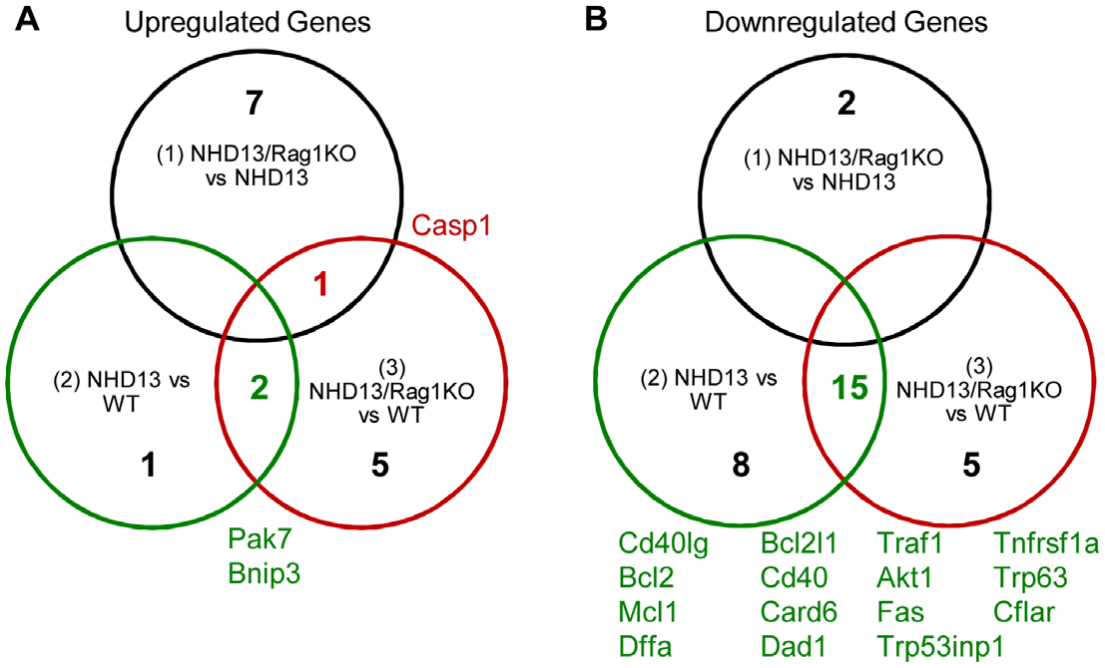
Differential expression of apoptosis pathway genes in T-ALLs of *NHD13* and *NHD13/Rag1*KO mice. Venn diagram of genes 4-fold elevated (A) or decreased (B) in comparison groups (1) *NHD13/Rag1*KO T-ALL vs *NHD13* T-ALL, (2) *NHD13* T-ALL vs WT Thy and (3) *NHD13/Rag1*KO T-ALL vs WT Thy. *Casp1* is common to group (1) and (3), and *Pak7* and *Bnip3* common to (2) and (3). The genes listed in (B) are common to groups (2) and (3).

We sought to identify alternative survival signals in the *NHD13/Rag1*KO pre-T-ALL by assessing the expression levels of genes known to be involved in apoptotic pathways. We used Mouse Apoptosis RT^2^ Profiler PCR Arrays to compare the expression levels of 84 genes involved in programmed cell death using pre-T-ALL samples from *NHD13/Rag1*KO and *NHD13* mice, and wild type thymus controls. We reasoned that the best candidate genes for an alternative survival signal would be increased in *NHD13/Rag1*KO pre-T-ALL compared to WT thymus, and also increased in *NHD13/Rag1*KO pre-T-ALL compared to *NHD13* pre-T-ALL (Figure 4a). However, *Casp1*was the sole gene identified in this screen, which was a poor candidate as an alternative survival signal given its role in promoting apoptosis [37]. We next identified genes that were overexpressed in both the *NHD13/Rag1*KO pre-T-ALL as well as the *NHD13* pre-T-ALL, reasoning that the alternative survival signal may be common to the *NHD13* background. This second screen identified the anti-apoptotic *Pak7* gene (20-fold increased) as the most dramatically overexpressed gene in the groups compared (Table 3). *Bnip3* (5.6-fold increased) was also overexpressed, but as a pro-apoptotic gene [38], [39], [40] appears to be an unlikely candidate for an alternative survival signal. There were no genes which were downregulated in the *NHD13/Rag1*KO pre-T-ALL compared to WT thymus, and also downregulated in the *NHD13/Rag1*KO compared to the *NHD13* pre-T-ALL. However, 15 downregulated genes were common to both the *NHD13/Rag1*KO pre-T-ALL vs WT Thy and *NHD13* pre-T-ALL vs WT Thy differential gene expression comparisons (Figure 4b). The downregulation of the pro-apoptotic genes *Dffa, Fas, Trp53inp1, Trp63*, and *Tnfrsf1* suggests that these genes might also promote the survival of the pre-T-ALL cells.

**Table 3:** Differential gene expression levels of apoptosis-related genes in WT thymus, and *NHD13* and *NHD13/Rag1*KO thymic tumors.

*NHD13/Rag1KO* vs *NHD13*			*NHD13* vs WT			*NHD13/Rag1KO* vs WT		
	Gene	FC		Gene	FC		Gene	FC
Increased	*Cd40lg*	9.64	Increased	*Pak7*	21.74	Increased	*Pak7*	19.85
	*Tnfsf10*	5.85		*Il10*	6.42		*Cidea*	11.60
	*Dffb*	5.63		*Bnip3*	4.79		*Casp1*	6.37
	*Trp73*	4.73					*Rnf7*	5.88
	*Trp63*	4.50					*Bnip3*	5.60
	*Fadd*	4.25					*Tsc22d3*	5.54
	*Birc5*	4.23					*Lhx4*	5.05
	*Casp1*	4.02					*Casp2*	4.14
Decreased	*Il10*	10.18	Decreased	*Cd40lg*	215.09	Decreased	*Cd40lg*	22.30
	*Casp7*	5.73		*Trp63*	41.44		*Bcl2l1*	20.66
				*Bcl2l1*	24.95		*Traf1*	20.31
				*Traf1*	13.63		*Tnfrsf1a*	19.21
				*Fas*	12.81		*Bcl2*	18.11
				*Cd40*	11.97		*Cd40*	13.45
				*Card6*	11.92		*Akt1*	9.31
				*Tnfrsf1a*	10.20		*Trp63*	9.22
				*Trp53inp1*	9.82		*Mcl1*	9.03
				*Tnfrsf10b*	7.18		*Cd70*	8.42
				*Dffa*	6.69		*Card6*	8.22
				*Bok*	5.89		*Trp53inp1*	7.41
				*Mcl1*	5.43		*Fas*	6.47
				*Dad1*	5.32		*Bad*	6.21
				*Fadd*	5.21		*Cflar*	5.93
				*Pim2*	4.75		*Ltbr*	5.85
				*Bcl2*	4.70		*Nol3*	5.04
				*Atf5*	4.39		*Dffa*	4.80
				*Cflar*	4.32		*Dapk1*	4.62
				*Akt1*	4.24		*Dad1*	4.53
				*Zc3hc1*	4.16			
				*Birc2*	4.10			
				*Nod1*	4.07			

FC, fold change.

## Discussion

Expression of a *NUP98-HOXD13* fusion gene in mice, has been shown to recapitulate the key features of human MDS, including peripheral blood cytopenias, dysplasia, increased apoptosis, ineffective hematopoiesis, and transformation to AML [21], [25], [26], [27]. However, none of these features differed between *NHD13* and *NHD13Rag1*KO mice, which lack T and B lymphocytes, demonstrating that an autoimmune mechanism is not involved in the form of MDS produced by the *NHD13* fusion gene, and therefore does not reflect the biology of immunosupression-responsive bone marrow failure that occurs in some MDS patients.

The *NHD13* mice displayed a modest (median 13 months vs 11 months) survival advantage compared to the *NHD13/Rag1*KO mice (p = 0.01). There was a non-significant trend toward an increased incidence of death due to leukemic transformation, as opposed to severe MDS, in the *NHD13/Rag1*KO compared to the *NHD13* mice. Somewhat surprisingly, there was no obvious difference in the proportion of lymphoid and myeloid leukemias in the *NHD13/Rag1*KO compared to the *NHD13* mice. There were no obvious differences in clinical presentation, gross necropsy findings, immunohistochemistry, or flow cytometry findings in the AML that developed in the *NHD13/Rag1*KO mice compared to the *NHD13* mice. Since *Rag1* deficient mice have far fewer immature T and B cells than do WT mice, we had anticipated that the *NHD13/Rag1*KO would have far fewer T or B cell leukemias compared to the *NHD13* mice. However, the B- and T-ALLs that developed in the *NHD13/Rag1*KO mice involved immature B- and T-cell precursors, consistent with previous reports of B-ALL[41], [42] and T-ALL[43] in *Rag1*KO mice. Similar to the lymphoid maligancies that arose in the *NHD13/Rag1*KO mice, the lymphoid leukemias that arose in the reports cited above all developed in the context of engineered mutations, such as deletion of *p19Arf*, *Trp53*, or overexpression of *Myc*.

There was a minor but significant survival disadvantage of the *NHD13/Rag1*KO mice compared to the *NHD13* mice (Figure 2A). Although it is possible that this survival disadvantage was due to infections of the immunodeficient *NHD13/Rag1*KO mice, we saw no findings on necropsy that would support this possibility, and there was no difference in survival of *Rag1*KO compared to WT mice (Figure 2A), which were housed in a Specific Pathogen Free (SPF) environment. It is possible that the improved survival was due in part to an immuno-surveillance mechanism (reviewed in [44]) present in the *NHD13* mice but lacking in the *NHD13/Rag1*KO mice. The absence of T- and B-cells, which normally mediate cellular immunity, transplant rejection, and elimination of nascent malignancies through antigen recognition, cytokine production, and T-cell activation, may allow progression of MDS to AML. This hypothesis would be supported by the trend toward increased AML transformation in the *NHD13/Rag1*KO mice compared to the *NHD13* mice (Figure 2B). However, given that the survival advantage was only two months, the effect is modest. Of note, a similar moderate survival advantage was observed in crosses of *Rag1*KO mice to other oncogenic backgrounds, such as those described above[41], [42], [43]. Interestingly, a *RAG1^−/−^* genotype also denotes a high-risk group with poorer survival in human B-ALL, again supporting the hypothesis that immune-mediated surveillance may be important in the elimination of fully transformed malignant cells [45], [46].

Given that the pre-T-ALL which arose in the *NHD13/Rag1*KO mice did not have *Tcrb* gene rearrangements, Tcrb could not have provided a survival signal to the malignant T cells. We used apoptosis focused gene arrays in an attempt to identify alternative candidates that may have provided a survival signal. *Pak7*(*Pak5*) was found to be upregulated 20-fold in the pre-T-ALL that developed in the *NHD13/Rag1*KO mice, and is an attractive candidate for such a survival signal. *PAK7* is normally expressed in the brain and is a member of the p21-activated kinase (PAK) family of proteins that act as upstream regulators of the MAPK signaling pathway[47]. TCR signaling normally activates JNK, p38 and NF-κB signaling pathways to instigate T-cell survival and activation (reviewed in [48]). Overexpression of *PAK7* activates the JNK kinase pathway[47] and can protect cells from apoptosis[49], [50], [51] thereby providing a viable survival stimulus in the absence of TCR. Various members of the PAK kinase family have been found to be overexpressed in cancers of the esophagus, breast, colon, liver, kidney, ovary, prostate, bladder, pancreas and brain, in neurofibromatosis, and in T-cell lymphoma (reviewed in[52], [53]). Taken together, although further studies would be required to prove that overexpression of *Pak7* provides a critical survival signal for the *NHD13/Rag1*KO pre-T-ALL, *Pak7* is a candidate for this function.

The findings presented here demonstrate the MDS which develops in *NHD13* mice is not dependent on an intact immune system, and is therefore not dependent on cytotoxic T-cells. Surprisingly, the *NHD13/Rag1*KO mice were not protected against lymphoid leukemias, and had a decreased survival compared to the *NHD13* only mice. An alternative survival signal for the pre-T-ALL that developed in the *NHD13/Rag1*KO may have been the marked overexpression of *Pak7* seen in these mice. Given that MDS is a heterogeneous disease, it is important to note that, although cytotoxic T-cells do not play a role in the development of MDS caused by a *NHD13* transgene, these findings do not preclude an effect of cytotoxic T-cells in MDS caused by other mechanisms.
